# Parental willingness to vaccinate their daughters against human papilloma virus and its associated factors in Woldia town, Northeast Ethiopia

**DOI:** 10.3389/fgwh.2024.1243280

**Published:** 2024-07-10

**Authors:** Sisay Melese Bittew, Seteamlak Adane Masresha, Getahun Fentaw Mulaw, Mohammed Ahmed Yimam, Abiot Alebel Zimamu, Atnaf Alem Abriham, Atitegeb Abera Kidie

**Affiliations:** ^1^ Woldia Comprehensive Specialized Hospital, Woldia, Ethiopia; ^2^ Department of Public Health, College of Health Science, Woldia University, Woldia, Ethiopia; ^3^ Gubalafto Woreda Health Office, Woldia, Ethiopia

**Keywords:** HPV, daughters, Northeast Ethiopia, parental willingness, North Wollo Zone

## Abstract

**Background:**

The cells of the cervical epithelial wall are the source of the malignant tumor caused by the human papilloma virus (HPV) known as cervical cancer. In 2018, Ethiopia implemented the HPV vaccine specifically targeting girls aged 9–14 years. This vaccination initiative serves as an effective preventive measure against cervical cancer, provided that parents express a positive inclination to have their daughters vaccinated as part of the program.

**Objective:**

The aim of the study was to assess parental willingness to vaccinate their daughters against human papillomavirus and its associated factors in Woldia town, Northeast Ethiopia.

**Methods:**

A community-based cross-sectional study was conducted among 414 parents of daughters aged 9–14 years between 10 and 25 January 2023. Respondents were selected by a systematic sampling method and a face-to-face interview was conducted to collect data. Data were entered into Epi Data version 4.6 and exported to SPSS version 25 for analysis. Multivariable analyses were used to examine the association between dependent and independent variables. The adjusted odds ratio (AOR), 95% confidence interval (CI), and *p*-value <0.05 were used to determine statistical significance.

**Results:**

A total of 410 study participants with a response rate of 99% were included in the study, and approximately 72.9% (95% CI: 68.3–77.2) of them were willing to vaccinate their daughters. This study found that parents with a family history of cervical cancer screening (AOR = 3.27, 95%; CI = 1.38–7.74), secondary and above educational status (AOR = 2.72, 95% CI = 1.29–5.73), good knowledge of the human papilloma virus vaccination (AOR = 3.00, 95% CI = 1.70–5.28), and favorable attitude toward the human papilloma virus vaccine (AOR = 4.40, 95% CI = 2.45–7.88) were significantly associated with parental willingness to vaccinate their daughters against human papilloma virus.

**Recommendation:**

In this study, most parents were willing to vaccinate their daughters against human papilloma virus. The significant determinants of parental willingness to their daughter's human papilloma virus vaccination were family history of cervical cancer screening, level of education, and knowledge and attitude toward the human papilloma virus vaccine. Therefore, health information regarding the human papillomavirus vaccination with an emphasis on raising community awareness should be designed.

## Introduction

The cells of the cervical epithelial lining are responsible for the malignant tumors also known as cervical cancer (CC). Cervical cancer is known to be caused by the sexually transmitted disease (STD) human papilloma virus (HPV), particularly the HPV 16, 18, and 31 serotypes (1). Cervical cancer is a malignant disease of the cervix that occurs at a mean age of 54 years; with a pre-malignant stage that usually occurs in younger women below the age of 40 years (2). More than 80% of women who are sexually active have been infected with HPV at least once in their lifetime (3).

Cervical cancer is the fourth most common type of diagnosed and lethal cancer in the world (4). Global estimates for 2020 showed there were 604,000 new cases and 342,000 fatalities each year (5). More than 80% of the cases are found in developing countries (6). The highest rates of death and incidence are found in sub-Saharan Africa, accounting for more than 70% of the cervical cancer burden in the world (5, 7). In Eastern Africa in 2018, there were 52,633 cases of cervical cancer (8). It is now the second most common cancer-related cause of mortality in Ethiopian women, accounting for more than 7,600 new cases and more than 6,000 fatalities each year (9).

Strategies for the prevention and control of cervical cancer include vaccination programs, screening, and treatment with standard of care (10). In many high-income countries (HICs), several primary and secondary preventive measures are available to anticipate the majority of fatal cancer developments (11). Low- and middle-income countries (LMICs) lack the resources for screening, early detection, and successful treatment of precancerous cervical lesions, which is an indicator as to why the provision of vaccinations is fundamental, especially in these regions (12). High-grade precancerous lesions, aggressive malignancy, and HPV infections can all be avoided with HPV vaccines (13). Comprehensive strategies and guidelines are created by the World Health Organization (WHO), including the HPV vaccination as the primary prevention for approximately 90% of CCs (14, 15). The WHO, the Centers for Disease Control and Prevention (CDC), the European Centers for Disease Prevention and Control (ECDC), and the American Academy of Pediatrics (AAP) recommend HPV vaccination for girls aged 11–12 years and a catch-up vaccination for girls aged 13–18 years, regardless of sexual activity (16). Global coverage of the HPV vaccination was 39.7%, including high-income countries (68%), middle-income countries (28%), low- to middle-income countries (2.7%), and Ethiopia (<2%) (17). The Global Alliance for Vaccine and Immunization (GAVI) has provided Ethiopia with access to the HPV vaccine since 2018. It is delivered via a method centered on schools (18). In Ethiopia, misconceptions about the cause and vaccination of cervical cancer are common because of a lack of awareness and health-seeking behavior (18). Nevertheless, research revealed that unfounded allegations regarding the vaccine’s adverse effects had a negative influence on public trust and caused the HPV vaccine program to be suspended (19, 20). As a result of the national scale-up of the CC prevention program, assessing barriers for parental willingness and using the service through appropriate community studies is critical. Since the HPV vaccine targeted young adolescent girls, the success of the HPV vaccination depends on parental decisions and their willingness to vaccinate their daughters (21). There are numerous types of literature on the Ethiopian context that explore the prevalence of cervical cancer and its risk factors (22); however, parental willingness to give their daughters the HPV vaccination is not well assessed in many LMICs, which is worse in Ethiopia (12).

Empirical evidence about parents' willingness to vaccinate their daughter against HPV is necessary as it is affected by different factors, such as poor knowledge of the HPV vaccination, lack of information sources, low household monthly income, negative rumors about the vaccine’s side effects, and illiteracy (16, 23, 24). As a result, it can cause vaccine refusal by parents for their daughters (25). Therefore, this study intended to assess parental willingness to vaccinate their daughters against HPV and its associated factors in Woldia town, Northeast Ethiopia.

## Methods

### Study design, area, and population

The study was conducted among parents of daughters who resided in Woldia town, which is located in the North Wollo Administrative Zone of Amhara regional State, Ethiopia.

A community-based cross-sectional study was carried out between 10 and 25 January 2023. Parents who had daughters aged 9–14 years in the selected kebeles of the town were the study population. Parents who had daughters aged 9–14 years and permanently residing in the study area (for ≥6 months) at the time of data collection were included. Parents whose daughters had previous or current marital history, had been pregnant previously, and/or were found to be pregnant at the time of data collection were excluded. A total of 414 respondents were selected using a multi-stage sampling technique.

Initially, kebeles were stratified as urban and rural, of which 50% of the total kebeles (three urban and two rural) were selected using the lottery method. Based on the data obtained from Woldia administrative town health department, the total number of households with eligible daughters in the town and selected Kebeles was 7,067 and 3,484, respectively. A proportional-to-sample size allocation (PSA) was then employed to determine the study participants from the selected kebeles. Next, 414 households were selected using a systematic sampling technique. One parent per household was interviewed. If both parents were present in the selected households, one was randomly chosen as a study participant. A structured face-to-face interviewer-administered questionnaire was used for data collection, which was developed after a thorough literature review of previously conducted studies. The questions for assessing knowledge and attitude were from previously validated tools of studies conducted in Ethiopia (18, 22, 24, 26).

The reliability of these questions was again assessed in this study and the reliability coefficient for each scale of the data collection tool revealed Cronbach alpha values of 0.95 and 0.74 for attitude and knowledge questioners, respectively.

The dependent variable of this study was parental willingness to their daughters’ HPV vaccination, which was categorized as yes/no. The independent variables of the study were sex, age, residence, religion, marital status, educational status, partner’s educational status, occupation, partner’s occupation, household monthly income, number of children, number of daughters (aged 9–14 years), family history of cervical cancer, family history of cervical cancer screening, family history of sexually transmitted infections (STIs), parental fear of STIs, daughters’ HPV vaccination history, source of information about HPV, trusted source of information, knowledge of the HPV vaccination, and attitude toward the HPV vaccine.

The terms used in this study were operationalized as follows:

***Attitude:*** Attitude toward HPV vaccination was assessed by using a 12-item attitude questions with five Likert scales ranging from strongly disagree to strongly agree with a maximum score of 12 * 5 = 60. Those respondents who scored greater than the mean value were categorized as having a favorable attitude, or otherwise categorized as an unfavorable attitude (22)*.*

***Knowledge:*** *Knowledge about the HPV vaccination was assessed using 15-item knowledge assessing questions* *with three responses: 1. Yes, 2. No, and 3. I do not know. Each correct and incorrect response scored 1 (yes = 1) and zero (no and I do not know = 0) points, respectively. The scores for each item were summed, and then those respondents who scored greater than the mean value were categorized as having good knowledge, otherwise poor knowledge* (22)*.*

### Data management and analysis

The collected data were entered into Epi Data version 4.6 and then exported to the SPSS version 25 for analysis. The findings were summarized and presented by tables, graphs, and other summary measures. Binary logistic regression was employed for final analysis. In the bi-variable logistic regression analysis, all independent variables with a *p*-value <0.25 were candidates for multivariable logistic regression model. An explanatory variable with a *p*-value <0.05 in the multivariable logistic regression analysis was considered significantly associated with the outcome variable. The model goodness of fit was assessed using the Hosmer and Lemeshow test and the model was well-fitted (*p*-value = 0.57). The presence of multi-collinearity between independent variables was checked by examining the standard error of regression coefficients for each independent variable. The strength of association was measured by adjusted odds ratio (AOR) with corresponding 95% confidence interval (CI) and those variables with a *p*-value <0.05 were considered statistically significant factors for the outcome variable.

## Results

### Sociodemographic and economic characteristics of the respondents

Out of 414 participants, 410 responded completely to the interview and yielded a response rate of 99%. More than three-quarters [3,140 (76.6%)] of the respondents were women. More than one-third [161 (39.3%)] of respondents were aged over 40 years, with a mean age of 38.6 ± 12.7 years. The majority [302 (73.6%)] of the respondents were married, 215 (52.4%) resided in urban areas, 273 (66.6%) had five or fewer children, and 360 (87.8%) had a daughter aged 9–14 years. Almost half [197 (48%)] of the study participants had a primary education and almost one-third [128 (31.2%)] of the respondents were housewives. Of the study respondents, more than half [226 (55%)] of them had a monthly income of less than 3,200 ETB (Table 1).

**Table 1:** Sociodemographic and economic characteristics of parental willingness to their daughters’ HPV vaccination in Woldia town, Northeast Ethiopia.

Variables	Categories	Frequency	%
Sex	Female	314	76.6
	Male	96	23.4
Age (years)	18–29	115	28.
	30–39	134	32.7
	≥40	161	39.3
Residence	Urban	215	52.4
	Rural	195	47.6
Marital status	Never married	20	4.9
	Married	302	73.6
	Divorced	59	14.4
	Widowed	29	7.1
Parents educational status	Unable to read and write	89	21.7
	Primary education	197	48.
	Secondary and above education	124	30.3
Partner educational status	Unable to read and write	82	27
	Primary education	115	38
	Secondary and above education	105	35
Religion	Orthodox	280	68.3
	Muslim	84	20.4
	Protestant	31	7.6
	Other^a^	15	3.7
Occupation	Civil servant	59	14.5
	Self-employed	85	20.7
	Merchant	32	7.8
	Farmer	78	19
	Housewife	128	31.2
	Other^b^	28	6.8
Partner occupation	Civil servant	72	23.8
	Self-employed	74	24.5
	Merchant	52	17.3
	Farmer	58	19.2
	Housewife	31	10.3
	Other^b^	15	4.9
Household monthly income (ETB)	<3,200	226	55
	≥3,200	184	45
Number of children	≤5	273	66.6
	>5	137	33.4
Number of daughters aged 9–14 years old	1	360	87.8
	≥2	50	12.2

ETB, Ethiopian birr.

^a^ Catholic and Yalem birhan.

^b^ Private employees, daily laborers, and working in non-governmental organizations.

### Reproductive health-related characteristics of the parents

Most [377 (92%)] of the study participants had no family history of cervical cancer, 340 (82.9%) participants had no family history of cervical cancer screening, 243 (59.3%) did not fear STIs, 234 (57.1%) had a family history of STIs, and half of the study participants’ daughters had no history of HPV vaccination (Table 2).

**Table 2:** Reproductive health characteristics of parental willingness to their daughters’ HPV vaccination in Woldia town, Northeast Ethiopia.

Variables	Categories	Frequency	%
Family history of cervical cancer	Yes	33	8
	No	377	92
Family history of cervical cancer screening	Yes	69	16.8
	No	341	83.2
Family history of STI	Yes	234	57.1
	No	176	42.9
Parental fear of STI	Yes	167	40.7
	No	243	59.3
Daughter's HPV vaccination history	Yes	204	49.8
	No	206	50.2

### Information, knowledge and attitude related to HPV vaccination

The majority [237 (57.8%)] of study participants had heard about the HPV vaccination. Regarding source of information, 97 (40.9%) participants heard from television and/or radio and 51 (21.5%) heard from health extension workers. Of the participants who had information about the HPV vaccination, television and radio were the trusted sources for more than half [138 (58.2%)] of the respondents followed by health providers [49 (20.7%)]. The majority [286 (69.8%)] of the study participants had poor knowledge of the HPV vaccination and 302 (73.7%) had a favorable attitude toward the HPV vaccination (Table 3).

**Table 3:** Source of information, attitude, and knowledge about HPV vaccination among parents of daughters in Woldia town, Northeast Ethiopia.

Variables	Categories	Number (*N*)	%
Having HPV vaccination information	Yes	237	57.8
	No	173	42.2
Source of information	Friends/relatives	22	9.3
	Television/radio	97	40.9
	Social media/internet	14	5.9
	Health extension workers	51	21.5
	Health professionals	30	12.7
	Newsletter/newspaper	12	5.1
	Other^a^	11	4.6
Trusted source of information	Friends/relatives	7	3.0
	Television/radio	138	58.2
	Social media/Internet	9	3.8
	Health extension workers	29	12.2
	Health professionals	49	20.7
	Newsletter/newspaper	3	1.3
	Other^b^	2	0.8
Knowledge of HPV vaccination	Poor	124	30.2
** **	Good	286	69.8
Attitude toward HPV vaccination	Unfavorable	108	26.3
** **	Favorable	302	73.7

^a^ Community volunteers, their children, groceries, mini media, and mobile message.

^b^ Mobile message and community volunteer.

### Parental willingness to daughters’ HPV vaccination

**Figure 1:**
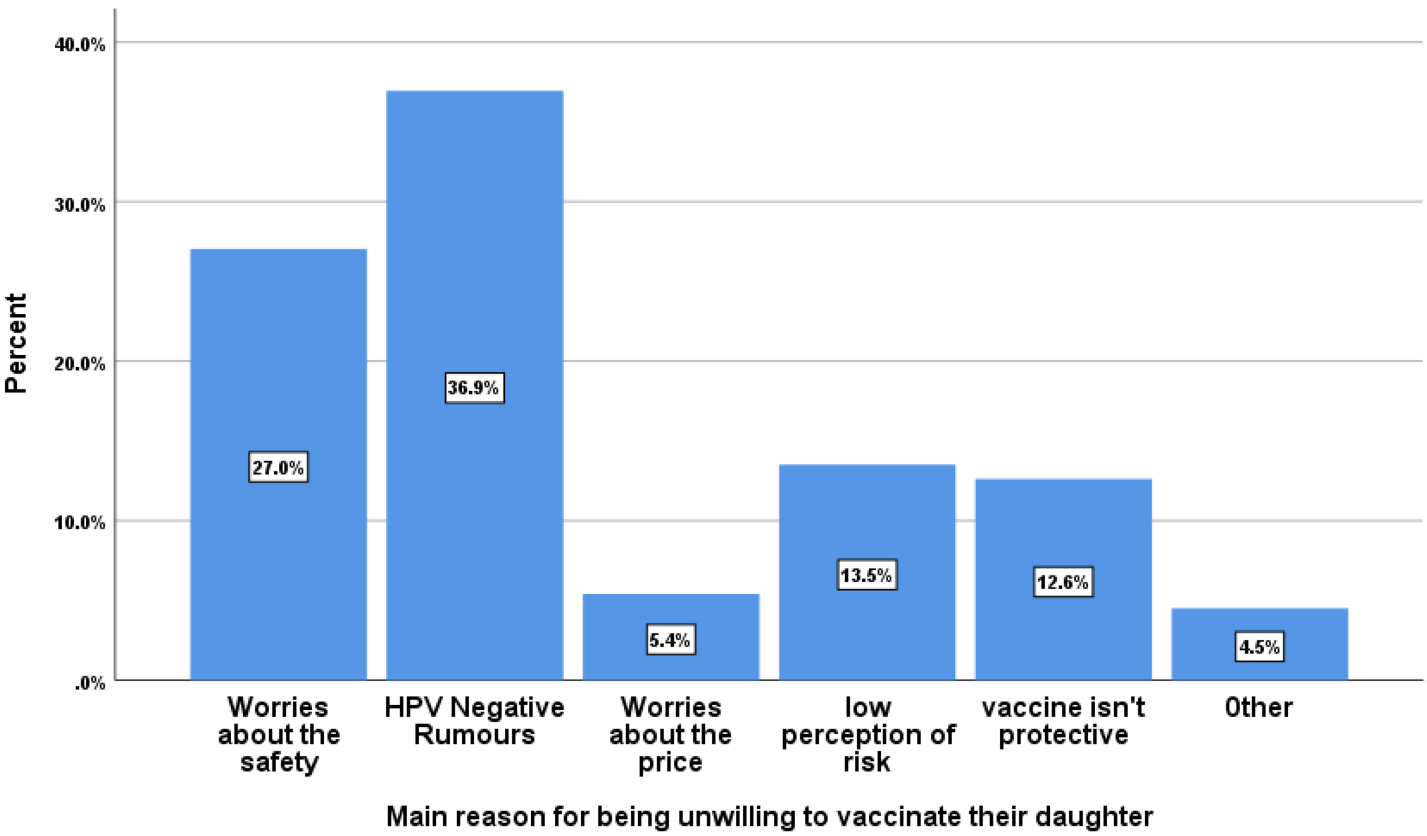
Main reasons for parental unwillingness toward the HPV vaccination in Woldia town, Northeast Ethiopia. Other = due to medical reasons or cultural and religious beliefs.

Out of 410 interviewed parents, 299 (72.9%, 95% CI: 68.3–77.2) were willing to vaccinate their daughters against HPV infection. The major reasons for not willing to vaccinate their daughters were considering the HPV vaccine as not being widely accepted (negative rumors about the vaccine) (37%), being worried about safety (feared side effects and perceived injection pain) (27%), low personal perception of risk for cervical cancer (13.5%), and considering the vaccine not protective (12.6%) (Figure 1).

### Factors associated with parental willingness to daughters' HPV vaccination

In the bi-variable analysis, variables such as age, number of children, number of daughters aged 9–14 years, family history of cervical cancer, family history of cervical cancer screening, family history of STIs, parents’ fear of STIs, daughter's HPV vaccination history, parents’ information about HPV, parents’ educational status, parents’ attitude and knowledge status about the HPV vaccination were eligible for a multivariable analysis at a *p*-value <0.25.

In the multivariable logistic regression analysis, parents with a family history of cervical cancer screening, secondary and higher education, good knowledge of and favorable attitude toward the HPV vaccination were significant factors for parental willingness for their daughter’s HPV vaccination at a *p*-value <0.05 (Table 4).

**Table 4:** Factors associated with parental willingness to their daughters’ HPV vaccination in Woldia town, Northeast Ethiopia.

Variables	Categories	Parental willingness to daughters’ HPV vaccination		COR (95% CI)	AOR (95% CI)
		Yes: *N* (%)	No: *N* (%)		
Age (years)	18–29	79 (26.4)	36 (32.4)	1	1
	30–39	99 (33.1)	35 (31.6)	1.28 (0.74–2.23	1.13 (0.57–2.21)
	≥40	121 (40.5)	40 (36)	1.37 (0.81–2.34)	1.68 (0.87–3.22)
Parents educational status	Cannot read and write	92 (19)	35 (28.8)	1	1
	Primary education	96 (45.5)	39 (55)	1.25 (0.73–2.12)	1.33 (0.72–2.46)
	Secondary and above	38 (35.5)	15 (16.2)	3.30 (1.70–6.40)	**2.72 (1.29–5.73)**
Number of children	≤5	204 (68.2)	69 (62.2)	1.30 (0.83–2.05)	1.39 (0.76–2.51)
	>5	95 (31.8)	42 (37.8)	1	1
Number of daughters (9–14 years old)	1	266 (89)	94 (84.7)	1.45 (0.77–2.73)	1.56 (0.72–3.39)
	≥2	33 (11)	17 (15.3)	1	1
Family history of CC	Yes	27 (9)	6 (5.4)	1.73 (0.69–4.32)	0.78 (0.26–2.33)
	No	272 (91)	105 (94.6)	1	1
Family history of CC screening	Yes	61 (20.4)	8 (7.2)	3.30 (1.52–7.14)	**3.27 (1.38–7.74)**
	No	238 (79.6)	103 (92.8)	1	1
Family history of STI	Yes	176 (58.9)	58 (52.3)	1.30 (0.84–2.02)	1.48 (0.81–2.70)
	No	123 (41.1)	53 (47.7)	1	1
Parental fear of STI	Yes	127 (42.5)	40 (36)	1.31 (0.83–2.05)	0.77 (0.39–1.52)
	No	172 (57.5)	71 (64)	1	1
Daughter's HPV vaccination history	Yes	154 (51.5)	50 (45)	1.29 (0.83–2.00)	1.11 (0.58–2.10)
	No	145 (48.5)	61 (55)	1	1
HPV information	Yes	178 (59.5)	59 (53.2)	1.29 (0.83–2.01)	0.73 (0.42–1.29)
	No	121 (40.5)	52 (46.8)	1	1
Knowledge of HPV vaccination	Poor	59 (19.7)	65 (58.5)	1	** **
	Good	240 (80.3)	46 (41.5)	5.74 (3.58–9.22)	**3.00 (1.70–5.28)**
Attitude toward HPV vaccine	Unfavorable	44 (14.7)	64 (57.6)	1	
	Favorable	255 (85.3)	47 (42.4)	7.89 (4.81–12.9)	**4.40 (2.45–7.88)**

*N*, frequency; COR, crude odds ratio.

Bold values indicate *p* < 0.05.

In this study, respondents who had a family history of cervical cancer screening were three times more likely to be willing to vaccinate their daughters compared to parents who had no family history of cervical cancer screening (AOR = 3.27, 95% CI = 1.38–7.74). Those parents with a secondary and higher education were three times more likely to be willing to vaccinate their daughter against HPV compared to those parents who were unable to read and write (AOR = 2.72, 95% CI = 1.29–5.73).

Parents who had good knowledge of the HPV vaccination were three times more likely to be willing to vaccinate their daughters compared to those with poor knowledge (AOR = 3.00, 95% CI = 1.70–5.28). Parents who had a favorable attitude toward the HPV vaccination were four times more likely to be willing to vaccinate their daughter than those who had an unfavorable attitude (AOR = 4.40, 95% CI = 2.45–7.88) (Table 4).

## Discussion

This study was carried out to determine parental willingness to vaccinate their daughters against HPV and its associated factors in Woldia town, Northeast Ethiopia. The study showed that nearly three-quarters (72.9%); [95% CI (68.3%, 77.2%)] of parents were willing to vaccinate their daughters for HPV infection. This study is in line with research conducted in the United Arab Emirates (76.6%) (27); Argentina (74%) (28); a study about parental acceptance of HPV in Lagos, Nigeria (72%) (29); and Gondar, Northwest Ethiopia (69.3%) (30). However, the results of this study are lower than those in studies carried out in South Africa (80%) (27); Poland (85.1%) (16); Tanzania (93%) (31); Lagos, Nigeria (81.8%) (32); Gondar, Northwest Ethiopia (81.3%) (22), and Addis Ababa, Ethiopia (94.3%) (33). As a result of the recent implementation of the HPV vaccination program in Ethiopia, the observed difference in vaccination rates could be attributed to concerns and negative rumors surrounding the potential side effects of the vaccine, as well as a lack of awareness regarding the personal risk of developing cervical cancer in the specific area under study. These findings are consistent with previous research that has identified similar factors as barriers to vaccination. This is supported by previous studies that reported similar reasons (24).

In addition, some of the above studies were carried out after the provision of HPV vaccines to daughters, which may improve parental willingness (27, 31, 32). The variation in the willingness rate from the study conducted in Addis Ababa could be due to the difference in the sociodemographic characteristics of the participants. Those living in Addis Ababa have greater access to health-based information, as expected, with a high level of health literacy compared to this study area, and there was a school-based HPV vaccination campaign in the city. Therefore, the parents might have heard and made their decision about the vaccination (33).

Conversely, the results of this study showed that there was a high level of parental willingness to vaccinate their daughters compared to those in previous studies conducted in Debre Tabor, Ethiopia (48.67%) (24) and Nigeria (67.4%) (34). The high vaccination acceptance rate may be attributable to the ongoing regional campaigns to raise awareness of cervical cancer and promote screening. The other reason in the present study, compared to the above studies, might be due to the current region-based cervical cancer awareness creation and screening campaign.

During the process of gathering data for this study, a campaign was launched in the town to raise awareness of the prevention of cervical cancer at the community level and cervical cancer screening service was being given in the town at the health facility level. The participants in the study may already possess knowledge about the HPV vaccination, which further increased their willingness (21). The high rate of willingness in this study area compared to Nigeria may be attributed to variations in the sociodemographic characteristics of the study participants (16). It might also be due to the introduction of the routine free HPV vaccination program at schools in this study area compared to Nigeria where vaccination is paid for out of pocket (18). The difference in the study period could contribute to the fact that earlier studies might represent periods when less information regarding the benefit of vaccines and the challenges posed by the disease was available (24, 34).

In this study, parents with secondary and higher educational status were more likely to be willing to vaccinate their daughters compared to participants who were unable to read and write. This finding of this study is similar to that in other studies conducted in the United States (USA) (35); China (36); Lagos, Nigeria (32); Alabama (37); Bench-Sheko zone, southwest Ethiopia (20); and Debre Tabor, Ethiopia (24). Parents that are better educated might have better access to information from schools, mass media, newspapers, and the Internet (24). Parents with higher levels of education might know about a specific disease and fear about their susceptibility to the disease, which creates health-seeking behavior that leads to an intention to understand the prevention of HPV infection. Thus, the educational level of parents significantly influences their willingness to vaccinate their daughters (19). However, apart from parental educational status, there was no significant association between other sociodemographic characteristics of respondents. This is supported by previous studies that reported similar findings (20, 37).

Parents with a family history of cervical cancer screening were more likely to be willing to vaccinate their daughters compared to parents who had no family history of cervical cancer screening. Parents who had a health facility visit for cervical cancer screening might have been exposes to cervical cancer counseling and important information on cervical cancer and HPV vaccinations. Therefore, this might create awareness, a favorable attitude toward the HPV vaccination, and the intention to understand the prevention of HPV infection, finally leading to willingness to vaccinate their daughters as well (38).

Parents with a good knowledge of the HPV vaccination were more likely to be willing to vaccinate their daughters against HPV than those with poor knowledge. This study finding is in line with those in previous studies conducted in Canada (39); Asia (40), Kilimanjaro Region, Tanzania (31); Gondar, Ethiopia (22); Bench-Sheko zone, southwest Ethiopia (20); Debre Tabor, Ethiopia (24); and Hadiya Zone, southern Ethiopia (41). This highlights that parental willingness to vaccinate their daughters against HPV is affected by their overall knowledge of HPV vaccination. This shows that knowledge of HPV and its vaccination triggers the participants to read and understand more about the route of transmission, consequence of infection, and complications of cervical cancer that may lead to behavioral change, which leads them to accept the HPV vaccination for their daughters (24, 42).

Parents who held a positive attitude toward HPV vaccination demonstrated a greater willingness to vaccinate their daughters against HPV infection compared to those who held a negative attitude. This is supported by study findings in South India (43); Iran (44); Gondar, Ethiopia (22); Bench-Sheko zone, Ethiopia (20); and Addis Ababa, Ethiopia (33). The reason for this could be attributed to the parents’ acceptance of the HPV vaccine, which is significantly influenced by their attitudes and beliefs regarding the vaccine's effectiveness, safety, and accessibility (14). Furthermore, the overall beliefs concerning the potential harm or benefits associated with the HPV vaccine can play a crucial role in determining a parent's decision to utilize the vaccine (23).

The major reasons why parents were not willing to vaccinate their daughters were considering the HPV vaccine not being widely accepted (negative rumors about the vaccine) (37%), being worried about safety (feared adverse effects and perceived injection pain) (27%) and having a low personal perception of risk for cervical cancer (13.5%). This is similar to other studies conducted in China, which indicated that approximately 43% of parents were worried about the safety and side effects of the HPV vaccine (45), in Nigeria, where 48.1% of parents were worried about adverse effects and 25.9% complained of poor availability of the vaccine (32), and in Debre Tabor, Ethiopia, where scarcity/cost of the HPV vaccine (57.4%), poor information about HPV vaccine (15.2%), undesirable impact on fertility (14.2%), and feared side effects (7.6%) were the reasons of parents for unwilling to vaccinate their daughters (24). A possible reason might be due to differences in sociodemographic characteristics, level of information, attitude, and knowledge status toward the HPV vaccination (29, 40).

### Strengths and limitations of the study

The strength of the study lies in its community-based study design, which used a representative sample of participants who were residents in both the urban and rural areas of Woldia town.

However, the study has some limitations. First, the study could not address “how” and “why” questions. In addition, it might be affected by desirability and recall bias since the data were collected from the participants’ self-report.

## Conclusion and recommendation

This study revealed that most parents were willing to vaccinate their daughters against HPV and it was significantly associated with family history of cervical cancer screening, parents’ level of education, parents’ knowledge of HPV vaccination, and parents’ attitude toward the HPV vaccination. Therefore, efforts should be made to increase awareness in the community of cervical cancer and its prevention to ensure sustainable parental willingness to the HPV vaccination. Specifically, educational campaigns should be conducted to disseminate information about cervical cancer, its causes, risk factors, and the importance of HPV vaccination. Collaboration with local healthcare providers would also be helpful to disseminate information about the prevention of cervical cancer and the HPV vaccination. Mixed studies are better for further investigations as they would provide a comprehensive understanding of why individuals may be unwilling to vaccinate their daughters. This approach can provide valuable insights into the “how and why” behind vaccine hesitancy among individuals, families, and communities, ultimately informing targeted strategies to address this important public health issue.

## Data Availability

The raw data supporting the conclusions of this article will be made available by the authors, without undue reservation.
